# Effect of Yogurt Addition on the Stability of Anthocyanin during Cold Storage of Strawberry, Raspberry, and Blueberry Smoothies

**DOI:** 10.3390/foods12203858

**Published:** 2023-10-21

**Authors:** Iwona Ścibisz, Małgorzata Ziarno

**Affiliations:** 1 Division of Fruit, Vegetable and Cereal Technology, Institute of Food Sciences, Warsaw University of Life Sciences WULS˗SGGW, 161 Nowoursynowska Str., 02-787 Warsaw, Poland; 2 Division of Milk Technology, Institute of Food Sciences, Warsaw University of Life Sciences WULS˗SGGW, 161 Nowoursynowska Str., 02-787 Warsaw, Poland; malgorzata_ziarno@sggw.edu.pl

**Keywords:** beverages, storage, stability, pigments, model experiment, pH, hydrogen peroxide, cell-free extract

## Abstract

The addition of yogurt to fruit smoothies enhances their nutritional value by introducing components not naturally found in fruit products. However, the addition of fermented products can affect the stability of fruit bioactive components in fruits, such as anthocyanins. This study aimed to evaluate the effect of varying yogurt additions (0, 10, 20, and 30%) on the stability of anthocyanins during a 4-week refrigerated storage period. The smoothies were obtained from purees of strawberry, raspberry, and blueberry, combined with apple juice and apple puree. In addition, to elucidate the causes of the observed changes in the smoothies, model studies were conducted using purified anthocyanin extracts obtained from the analyzed fruits. We assessed the effects of pH, hydrogen peroxide concentration, and the addition of cell-free extracts from *Streptococcus thermophilus* and *Lactobacillus delbrueckii* subsp. *bulgaricus* on changes in anthocyanin content during storage. We found that adding yogurt led to a decrease in anthocyanin stability during the 4-week cold storage period. Specifically, a 30% yogurt addition decreased anthocyanin stability in all tested beverages, while a 20% yogurt addition impacted the strawberry and raspberry smoothies. The degree to which yogurt affected anthocyanin stability was dependent on the source of the raw material. The most notable impact was observed in strawberry smoothies and the least in blueberry smoothies. The variability could be attributed to differences in anthocyanin profiles among the fruits, the chemical composition of the beverages, and the observed difference in the survival rates of lactic acid bacteria. Model studies showed that during the storage of anthocyanin extracts, the addition of hydrogen peroxide and cell-free extract had a significant effect, whereas pH within the examined range (3.0–4.5) did not affect anthocyanin stability.

## 1. Introduction

In recent years, consumers have become increasingly aware of the importance of proper nutrition and are paying more attention to the nutritional value of their products and beverages. Smoothies with plenty of fruit tissue, which are a rich source of fiber and antioxidant compounds, are becoming increasingly popular and are currently one of the major segments in the soft drinks market. The growth in sales of the smoothie market is driven by consumers with healthy lifestyles. Consequently, beverage manufacturers are focusing more on clean labeling and simple composition, without coloring substances or colored food [1,2].

A smoothie is a thick product with a smooth texture made by blending fruits and/or vegetables in the form of juice and puree in specific proportions. The primary ingredients often include popular fruits such as bananas, apples, oranges, strawberries, and blackberries. In addition to these common fruits, less prevalent options such as raspberries and blueberries are also used in smoothie production. These less common fruits are rich in polyphenolic compounds, including anthocyanins, which lend an attractive color to the smoothie. To diversify the assortment of beverages, producers use ingredients with high nutritional value that offer health benefits, such as milk, seeds, or yogurt. The addition of yogurt into a fruit smoothie increases the nutritional value of the product. Yogurt is a source of bioavailable calcium, provides proteins of high biological value and essential amino acids, and various vitamins including riboflavin, thiamin, and vitamin B_12_. Additionally, yogurt can introduce significant amounts of probiotic bacteria into the body, which are claimed to have specific health benefits [3,4,5].

However, a primary challenge in manufacturing fruit-based products that contain anthocyanins along with dairy yogurt is the low stability of these pigments during storage. The stability of anthocyanins is crucial not just for maintaining the color of the products, but also for their potential health benefits to consumers. Studies confirm the favorable role of anthocyanins in human health, such as preventing intracellular oxidation and acting as a protective agent against acute liver injury and cardiovascular disease [6]. Adding yogurt to fruit smoothies can potentially raise the pH, which affects the stability of anthocyanins. Many model studies suggest that a higher pH environment accelerates the degradation of these pigments during storage. However, small differences in the pH of the environment did not affect the stability of the anthocyanins [7,8]. In addition, Sasaki et al. (2014) [9] showed that *Lactobacillus delbrueckii* subsp. *bulgaricus*, a bacteria used in yogurt production, produces significant amounts of hydrogen peroxide during milk fermentation. Studies in the context of aseptic juice packaging or disinfection methods have shown that hydrogen peroxide has a destructive effect on anthocyanins. This impact varies depending on both the amount of H_2_O_2_ and the source of the anthocyanins [10].

Recent research indicates that lactic acid bacteria can produce β-glucosidase, which can hydrolyze the glycosidic bonds of anthocyanins into corresponding anthocyanidins, which are unstable and easily degraded [11]. Research comparing the β-glucosidase activity among different strains of lactic acid bacteria has shown that it depends not only on the bacterial strain but also on the pH and temperature of the environment [12]. Some recent studies have also reported that certain components in yogurt may enhance the stability of anthocyanins. Proteins, amino acids, and peptides have been shown to increase colorant stability in stored products by forming complexes [13,14]. Additionally, the fat content in yogurt has been identified as a stabilizing factor for anthocyanins during storage [15]. Many studies have been conducted to determine the stability of anthocyanins during the storage of fruit juices, demonstrating that stability depends on various factors such as the type of anthocyanin, the origin of the juice, and especially the storage temperature [16,17].

However, there are not many studies determining the stability of anthocyanins in fruit smoothies that contain added yogurt. Alsubhi et al. (2022) [18] found that incorporating pomegranate pomace extract stabilized the color of a strawberry–yogurt smoothie when stored under refrigerated conditions for 2 months. However, to the best of our knowledge, no published studies have specifically compared the loss of anthocyanins in fruit smoothies based on the proportion of yogurt added. Such studies could offer valuable insights for beverage formulation and furnish a comprehensive knowledge of the stability of compounds that are not only important for the product’s quality but also for its health benefits.

Therefore, the purpose of this study was to evaluate the effect of adding yogurt to strawberry, raspberry, and blueberry smoothies on the stability of anthocyanins during 4 weeks of refrigerated storage. Moreover, we evaluated the physicochemical parameters of the semifinished products used in the smoothie production. Changes in the count of lactic acid bacteria during storage were also assessed. To further understand the factors affecting anthocyanin stability in purified extracts, a model study was conducted. This model evaluated the influence of environmental pH, hydrogen peroxide concentration, and the addition of cell-free extracts from *Streptococcus thermophilus* and *L. delbrueckii* subsp. *bulgaricus*. The conditions for these model experiments were chosen to closely mimic those encountered during the storage of the smoothies.

## 2. Materials and Methods

### 2.1. Materials and Chemicals

Fruits of strawberry (*Fragaria* x *ananassa* Duch., cv. Senga Sengana), raspberry (*Rubus idaeus* L., cv. Polka), and highbush blueberry (*Vaccinium corymbosum* L., cv. Patriot) were sourced from fruit growers located near Warsaw, Poland. These fruits were stored frozen at −35 °C until they were processed into purees. Golden Delicious apples were obtained from the experimental orchard of the Faculty of Horticulture and Biotechnology at SGGW and stored at temperatures between 0 and 2 °C until the puree and juice production. Commercial UHT milk, with a fat content of 3.2%, was bought from a local market in Warsaw, Poland, and stored at 4 °C until use. A lyophilized culture of YC-X16, comprising *S. thermophilus* and *L. delbrueckii* subsp. *bulgaricus*, was purchased from Chr. Hansen (Hørsholm, Denmark).

L-ascorbic acid, dithiothreitol (DTT), Folin–Ciocalteu’s reagent, trichloroacetic acid, phosphoric acid, lactose, glucose, fructose, saccharose, galactose, and dianisidine were obtained from Sigma-Aldrich. Malic acid, citric acid and L(+)-lactic acid, and horseradish peroxidase were purchased from Merck KGAA (Darmstadt, Germany). Meta-phosphoric, methanol, and acetonitrile were obtained from Honeywell Fluka (Honeywell International, Inc., Charlotte, NC, USA). Cyanidin-3-O-arabinoside, malvidin-3-O-glucoside, and peonidin-3-O-glucoside were purchased from LGC Standards (Teddington, UK), delphinidin-3-O-glucoside, cyanidin-3-O-glucoside, pelargonidin-3-O-glucoside, cyanidin-3-O-sophoroside, and pelargonidin-3-O-rutinoside were obtained from Carbosynth Ltd. (Berkshire, UK) and cyanidin-3-O-galactoside was purchased from Extrasynthese (Lyon, France). All solvents used were of analytical or HPLC grade.

### 2.2. Production of Smoothie

The smoothie production process involved the production of fruit semiproducts, including apple juice and purees of strawberry, raspberry, blueberry, and apple. These semiproducts were then mixed in varying ratios to produce smoothies with 0, 10, 20, and 30% yogurt content (Table 1). All semiproducts were prepared in duplicate. For puree production, strawberries (5 kg), raspberries (6 kg), and blueberries (6 kg) were thawed at room temperature, while apples (5 kg) were washed and quartered. The fruits were then heated—apples in a steamer and berries in a microwave—until the temperature at the geometric center of the material reached 85 °C. Following heating, the fruits were processed with a laboratory pulper equipped with a sieve having 0.8 mm diameter holes. The purees were further homogenized using a laboratory colloid mill (IKA Magic Lab, Staufen, Germany) under reduced pressure. Once heated to 70 °C, the purees were transferred to glass jars, sealed with plastic-lined metal caps, and pasteurized at 85 °C for 10 min. After pasteurization, the puree samples were cooled to 20 °C using chilled water and stored under refrigerated conditions until they were used in smoothie production (no longer than 2 weeks).

In the juice production process, 10 kg of apples were washed and then crushed using an apple shredder. The juice was subsequently extracted by pressing the mash with a laboratory press, yielding approximately 58 ± 7% juice. The extracted juice was poured into glass bottles (220 mL), sealed with plastic-lined metal caps, and pasteurized at 85 °C for 10 min. After pasteurization, the juice samples were cooled to 20 °C and stored in the dark at 2 °C for 1 week.

Yogurt was prepared using the tank method. The yogurt starter culture was initially hydrated with a small amount of UHT milk before being added to the main milk volume, following the manufacturer’s recommendations. Fermentation proceeded for about 4–5 h at 37 °C until the yogurt reached a pH of approximately 4.2. The fat and protein content of the obtained yogurt were tested. Analysis of the fat content was conducted following the guidelines outlined in ISO 19662:2018 Milk—Determination of fat content—Acido-butyrometric (Gerber method) [19] on three independent samples. Analysis of the protein content in the yoghurt samples was carried out following the guidelines specified in ISO 1871:2009 [20] Food and feed products—General guidelines for the determination of nitrogen by the Kjeldahl method on three independent samples. The final yogurt samples were also tested for the population of lactic acid bacteria: *L. delbrueckii* subsp. *bulgaricus* and *S. thermophilus*.

The purees, juice, and yogurt were combined according to the proportions outlined in Table 1 within a laminar flow cabinet. The resulting smoothies were poured into sterilized glass bottles (220 mL) and sealed with plastic-lined metal caps. In total, 12 different smoothies were created, each with the same quantity of strawberry, raspberry, or blueberry puree but varying amounts of yogurt, apple juice, and apple puree. For each smoothie type, 15 bottles were stored in the dark at 2 °C for 4 weeks. The anthocyanin content was monitored both at the onset of cold storage and at weekly intervals throughout the storage period. Samples of the semiproducts used in the smoothie production were also analyzed and stored in a freezer until further analysis (−35 °C).

### 2.3. Extraction and Purification of Anthocyanins from Fruits

The anthocyanins were extracted from the fruit following the method outlined by Veigas et al. (2007) [21], with some modifications. Initially, 200–400 g of strawberry, raspberry, or blueberry fruits were homogenized and extracted using 0.5 L of a methanol:water (700:300, *v*:*v*) solution containing 0.1% HCl. The extract was filtered through Whatman No. 1 paper, and the residue was re-extracted until the filtrate became nearly colorless. The extracts were then combined and concentrated at 40 °C using a rotary evaporator (Buchi, Switzerland) to remove the methanol. Subsequently, the obtained extract was partitioned with ethyl acetate (4 × 100 mL) using a separatory funnel. After evaporating the remaining ethyl acetate and performing centrifugation, the aqueous extract containing anthocyanins was loaded onto a column (3 cm × 40 cm) of Amberlite XAD-7HP cation-exchange resin (Sigma-Aldrich, Saint Louis, MI, USA). The resin was washed with 2 L of acidified water at a flow rate of 1 mL/min to remove free sugars, proteins, ions, and organic acids [22]. The adsorbed anthocyanins were then eluted from the column using 35% ethanol containing 0.01% HCl at a flow rate of 1.5 mL/min. Finally, the collected fraction was concentrated by evaporating the ethanol under vacuum using a rotary evaporator at a temperature of 40 °C and stored at −30 °C.

### 2.4. Model Experiments

The following model experiments were carried out to assess the storage stability of anthocyanins under varying pH levels, different concentrations of hydrogen peroxide, and in the presence of cell-free supernatant from yogurt bacteria monocultures.

pH model study. Citrate buffers were added to the extracted anthocyanins to achieve a final concentration of 15 mg/100 mL. The pH levels for these extracts were adjusted to 3, 3.5, 4, and 4.5. Buffers were prepared using solutions of 0.1 M citric acid and 0.1 M sodium citrate.

H_2_O_2_ model study. To a solution containing 15 mg/100 mL of fruit anthocyanins and citrate buffers at pH 4, varying concentrations of hydrogen peroxide were added to achieve final concentrations of 0, 1, 3, and 6 µg/mL. The volumes of these solutions were adjusted to be equal.

Cell-free extracts model study. Monocultures of *S. thermophilus* and *L. delbrueckii* subsp. *bulgaricus*, isolated from YC-X16 starter cultures, were cultivated at 37 °C for 24 h in M17 and MRS broths, respectively. Post-incubation, the broths were centrifuged at 13,000× *g* at 4 °C for 10 min to obtain cell-free extracts. The resulting cell-free supernatants were added to the anthocyanin solutions, maintaining a final anthocyanin concentration of 15 mg/100 mL. Citrate buffer at pH 4 was used to prepare these solutions.

The volume for all model anthocyanin extracts was set at 10 mL, with an initial anthocyanin concentration of 15 mg/100 mL. Ten groups of anthocyanin solutions derived from different fruits were prepared in tubes, each with three replicates. These were stored in the dark at 2 °C for 4 weeks, and the anthocyanin content was recorded weekly.

### 2.5. Analytical Methods

#### 2.5.1. The Physicochemical Analysis

Soluble solids were determined as °Brix using an electronic refractometer calibrated at 20 °C. pH was measured using a calibrated pH meter (HI221; Hanna Instruments, Smithfield, RI, USA). Total acidity was ascertained by titration with 0.1 N NaOH to a pH of 8.2 and was expressed as grams of citric, malic, or lactic acid per 100 g for red purees, apple products, and yogurt, respectively. Total phenolic compounds were determined according to the Folin–Ciocalteu method [23], with minor modifications. Gallic acid served as the standard, and the results were expressed as mg of gallic acid equivalents per 100 g. For the extraction of total phenolics in fruit products, a mixture of methanol/acetone/water (350:350:300) was utilized. In the case of yogurt, the pH of samples was adjusted to 4.6 with 1 M HCl, and nonhydrolyzed casein was removed via centrifugation [24].

#### 2.5.2. Determination of Sugars and Organic Acids

Chromatographic analyses were performed on a Shimadzu Prominence HPLC system (Kyoto, Japan), comprising an LC-20AD pump, degasser model (DGU-20A5R), and column oven, along with an autosampler (SIL-20A HT) and LABSolutions data collection software version 5.97. The sugar analysis in semiproducts followed the procedure described by Usenik et al. (2008) [25], with minor modifications. Puree samples were homogenized for a few seconds using a homogenizer (IKA Labortechnik, Germany). Approximately 5–10 g of the sample were diluted to 50 mL with distilled water. The solution was then stirred in a thermostat set at 25 °C (Unithermix, LLG Labware, Meckenheim, Germany), with frequent stirring at 250 rpm for 30 min. Post-extraction, the samples were centrifuged (MPW-350R; MPW Med. Instruments, Warsaw, Poland) for 10 min at 15,133× *g* and 4 °C to separate the layers. The resulting supernatants were filtered through a 0.45 µm filter and used for HPLC analyses. Sugar analysis was performed using the aforementioned Shimadzu HPLC system, equipped with a refractive index detector. Separation was executed on a RezexTM RCM-Monosaccharide Ca^+^ column (300 × 7.8 mm) (Phenomenex, Torrance, CA, USA) at 75 °C, employing Milli-Q water as the mobile phase and a flow rate of 0.6 mL/min.

For determining organic acid content, high-performance liquid chromatography was employed according to a modified method from Flores et al. (2012) [26]. Homogenized samples (2.5 g) were extracted with 10 mL of distilled water for 10 min at 25 °C, with stirring at 250 rpm. The samples were then centrifuged, and this extraction procedure was repeated. The resulting supernatants were combined to reach a final volume of 25 mL with water. Subsequently, the extracts were passed through a Sep-Pak C18 cartridge (Waters, Milford, MA, USA) to eliminate potential interferences, then filtered through a 0.45 µm filter and transferred into vials. Analysis was performed on a Shimadzu chromatographic system fitted with a diode array detector. Organic acid separation took place isocratically on a Cosmosil 5C18-PAQ (4.6 mm × 150 mm) column, using a mobile phase of 20 mmol phosphoric acid with a flow rate of 0.7 mL/min. Detection was carried out at a wavelength of 210 nm. Both organic acids and sugars were identified by comparing their retention times and UV–vis spectral characteristics (in the case of organic acids) to those of commercial standards.

#### 2.5.3. Vitamin C Determination

An HPLC system equipped with a diode array detector and an Onyx Monolithic (100 × 4.6 mm) column, along with an Onyx Monolithic C18 guard cartridge (10 × 4.6 mm; Phenomenex, Torrance, CA, USA), was utilized to measure the concentration of vitamin C. The method was adapted from Chebrolu et al. (2012) [27] with certain modifications. Dehydroascorbic acid was reduced to ascorbic acid by adding 10 mmol/L of dithiothreitol (DTT). A 2 g sample was extracted in 10 mL of 2% metaphosphoric acid. The extract was vortex-mixed for 10 s, centrifuged for 10 min at 15,133× *g* and 4 °C, and then filtered through a nylon 0.45 µm filter. In the case of yogurt, post-centrifugation, the sample was simply filtered through a syringe filter. For ascorbic acid analysis, 300 µL of the supernatant was mixed with an equal volume of 2% metaphosphoric acid and immediately injected into the HPLC system. For the analysis of both ascorbic and dehydroascorbic acids, the supernatant (300 µL) was combined with 300 µL of DTT solution; after 30 min, the sample was injected into the HPLC system. Chromatographic analyses were conducted using 0.1% phosphoric acid as the mobile phase, with a flow rate of 1 mL/min. Absorbance was monitored at 254 nm. The vitamin C content was expressed in mg per 100 g of semiproducts, representing the sum of L-ascorbic acid and L-dehydroascorbic acid found in the samples. All samples were prepared in triplicate.

#### 2.5.4. Determination of Hydrogen Peroxide Content

The content of hydrogen peroxide was determined by an enzymatic method, adapted from studies by Yap and Gilliland (2000) [28] and Zhou et al. (2006) [29]. Approximately 2–5 g of fruit semiproducts were homogenized with 10 mL of 1% trichloroacetic acid (*w*/*v*). The homogenates were then centrifuged at 15,133× *g* for 10 min. Following this, 0.2 g of insoluble polyvinylpolypyrrolidone and 0.3 g of activated charcoal were added, and the samples were centrifuged again at 15,133× *g* and 4 °C for an additional 5 min. Two milliliters of the supernatants were mixed with 3 mL of 0.1 M acetate buffer (pH 4.5).

For the yogurt sample, 10 mL of yogurt was adjusted to pH 4.5 using 0.1 N HCL. After this adjustment, 2 mL of acetate buffer (pH 4.5) was added, and the mixture was transferred to a 25 mL volumetric flask, which was then filled to volume with distilled water. These samples were centrifuged at 4075× *g* for 10 min. Next, 5 mL of the supernatant from both the yogurt and fruit samples were added to a glass tube containing 100 µL of 1% o-dianisidine and 1 mL of 1% horseradish peroxidase. The samples were mixed and incubated at 37 °C for 10 min. Subsequently, 0.2 mL of 4 N HCL was added to stop the reaction. After a 5-min wait, absorbance was recorded at 400 nm. The content of hydrogen peroxide was calculated by comparison to a standard calibration curve and expressed as µg per g of samples.

#### 2.5.5. Enumeration of Viable Bacteria

The number of viable bacterial cells during the storage of the smoothie was enumerated using the standard plate method [30]. Samples were serially diluted to the appropriate concentration using peptone water (1.0 g/L) and then poured onto specific growth media—MRS agar for *L. delbrueckii* subsp. *bulgaricus* and M17 agar for *S. thermophilus*. Viable counts were determined after 72 h of anaerobic incubation at 37 °C for *L. delbrueckii* subsp. *bulgaricus* and aerobic incubation for *S. thermophilus*. The viable cell count was expressed as the log of colony-forming units per g (log CFU/g) of the smoothie.

#### 2.5.6. Analysis of Anthocyanins

For the extraction of anthocyanins from the fruit smoothie, a mixture of methanol, water, and hydrochloric acid (700/300/1, *v*/*v*/*v*) was used. A 20-g sample of the smoothie was stirred with 30 mL of the extraction mixture for 10 min at 25 °C (Unithermix; LLG Labware) and then sonicated for an additional 10 min (ultrasonic bath, SW 3H; Sonoswiss AG, Ramsen, Switzerland). The samples were subsequently centrifuged (6880× *g* for 10 min), and the supernatant was used for further assays. This extraction process was repeated three to five times until the sample was completely discolored. The obtained extract was stored at freezing temperatures to precipitate proteins and then centrifuged once more. Samples were evaporated using a rotary evaporator under reduced pressure at 40 °C until all methanol was removed, and then the volume was brought up to 50 mL using phosphoric acid (1.0 g/L). To purify the samples from sugars and organic acids, Sep-Pack® C18 cartridges (Waters, Milford, MA, USA) were used, which were preconditioned with 10 mL of methanol followed by 10 mL of 0.1% phosphoric acid. The extract (10 mL) was loaded onto the cartridge, followed by an additional 10 mL of 0.1% phosphoric acid, and was then eluted with 5 mL of methanol containing 0.1% HCl. The resultant samples were filtered through 0.45 µm PTFE syringe filters before undergoing chromatographic analysis. Anthocyanin extracts obtained from these model experiments were diluted with 0.1% phosphoric acid, filtered through syringe filters, and analyzed immediately.

The Shimadzu Prominence HPLC system mentioned earlier, equipped with a DAD detector (SPD M20A), was used to analyze the anthocyanins in the samples. Separation was conducted using a Luna C18(2) RP (5 m) 250 × 4.6 column, fitted with a KJO-4282 guard column (Phenomenex, Torrance, CA, USA). Anthocyanins were eluted through a gradient comprised of formic acid/water (mobile phase A, 100:900, *v*/*v*) and acetonitrile/formic acid (mobile phase B, 900:100, *v*/*v*). The flow rate was set at 1 mL/min. For strawberry and raspberry products, the gradient elution was as follows: 0–2 min, 9% B; 2–7 min, 9–20% B; 7–12 min, 20–50% B; 12–14 min, 50% B; 14–16 min, 50–9% B; followed by 9% B for 8 min. For blueberry products, the gradient was: 0–5 min, 5.5% B; 5–8 min, 5.5–9% B; 8–20 min, 9–11% B; 20–22 min, 11–14% B; 22–27 min, 14–22% B; 27–31 min, 22–35% B; 31–40 min, 35–5.5% B; 5.5% B for 7 min. The injection volume was 20 µL. Anthocyanins were identified by comparing their elution order and retention time to authentic standards or data reported previously [31]. Pelargonidin-3-O-glucoside (strawberry), cyanidin-3-O-glucoside (raspberry), or malvidin-3-O-glucoside (blueberry) were used as external standards for the quantification of anthocyanins. Anthocyanins were quantified at 520 nm and the content was expressed as mg/100 g of smoothie or 100 mL extract in model solutions.

### 2.6. Statistical Analysis

Analyses were conducted in triplicate, following a two-factor experimental design (addition of yogurt × storage time or, in the model system, conditions × storage time). This design was subjected to analysis of variance (ANOVA) using the Statistica 13.3 software package (Tibco Inc., Stafford, TX, USA). To examine differences between means, Tukey’s multiple range test was applied at a significance level of *p* ≤ 0.05.

## 3. Results and Discussion

### 3.1. Physicochemical Parameters of Semi-Products Used in the Production of Smoothies

In the fruit semiproducts used for smoothie production, soluble solids varied from 9.1°Brix in strawberry puree to 12.8°Brix in blueberry puree (Table 2). The fructose was the sugar found in the highest concentration in the semiproducts analyzed, and its content ranged from 3.3 g/100 g for strawberry puree to 6.1 g/100 g for apple puree. The products obtained from apples had the highest sucrose content, while this sugar was not detected in blueberry puree. Compared to previous work, the purees showed a lower sucrose content than that found in whole fruits. However, during the heat treatment used in the production of purees, sucrose may have been hydrolyzed into directly reducing sugars. Moreover, the sucrose content in fruit can also vary depending on the degree of ripeness and the conditions of postharvest storage [32,33]. The yogurt used in the smoothie production had a high lactose content (3.8/100 g) and low levels of galactose, with glucose levels close to the detection limit. A study by Ohlsson et al. (2017) [34] reported lower lactose but similar galactose levels in Swedish market yogurt. Differences in sugar composition could be attributed to factors such as fermentation conditions, the specific starter bacteria used in yogurt production (affecting the degree of enzymatic lactose hydrolysis), and the storage time of fermented products.

Titratable acidity, pH, and organic acid content are commonly measured as overarching quality indicators. These factors not only determine the preservation conditions of fruit products but also influence the stability of anthocyanins and the viability of lactic acid bacteria in dairy–fruit products [32,35,36]. The titratable acidity in fruit semiproducts ranged from 0.45 g/100 g in blueberry puree to 1.2 g/100 g in raspberry puree. A higher titratable acidity in these fruit products was associated with a lower pH and higher content of organic acids. Conversely, the yogurt exhibited relatively high acidity, yet its pH was the highest among all tested products. This can be attributed to the presence of proteins and phosphates in milk, which are known for their buffering effects [37]. The titratable acidity of the yogurt used in the production of smoothies was 0.76 g/100 g and the pH was 4.17. These results are similar to those reported for commercial and plain yogurts [38,39], but differ from those for yogurts fermented by *L. brevis* and *L. acidophilus* [37]. The final acidity of yogurt is influenced by several variables, the most significant of which are the type of starter cultures as well as the time and temperature of fermentation.

Fruits are a rich source of vitamin C and phenolic compounds, which are reported to have a range of potential anticancer and anti-heart disease benefits. Significant differences were observed in the vitamin C and polyphenolic content among the analyzed semiproducts. Strawberry puree had the highest vitamin C content, while blueberry puree had the higher amount of phenolics. Interestingly, significant differences were found in the total phenolic content between puree and juice obtained from the same apple variety. Such differences can be attributed to the oxidation reactions catalyzed by polyphenol oxidase during the crushing and pressing stages of juice production or the inefficient transfer of phenolic compounds from the apple mash to the juice. The yogurt had the lowest amounts of both vitamin C (0.3 mg/100 g) and total phenolic content (5.6 mg/100 g) among the tested semiproducts. These findings are consistent with previous research [37,40,41]. For the determination of total phenolic content, nonhydrolyzed casein was removed from the yogurt to prevent interference with spectrophotometric determination through reaction with the Folin–Ciocalteu reagent. However, the phenolic content in yogurt may be overestimated due to the presence of amino acids and whey proteins in the samples, which can also react with the Folin–Ciocalteu reagent.

The results showed that the highest content of hydrogen peroxide was determined in yogurt samples, amounting to approximately 5.9 µg/g. The presence of high levels of hydrogen peroxide in yogurt could be a contributing factor to the accelerated degradation of anthocyanins observed in yogurt-containing smoothies during storage. Hydrogen peroxide is a reactive oxygen species (ROS) known for its oxidative properties. It can facilitate the breakdown of anthocyanins, leading to their degradation and color loss. The high hydrogen peroxide content in yogurt is attributable to the presence of lactic acid bacteria, particularly the activity of *L. delbrueckii* subsp. *bulgaricus* used in production [42,43]. Numerous studies have confirmed that the presence of lactobacilli strains leads to high levels of hydrogen peroxide, mainly due to the action of NADH:H_2_O_2_ oxidase, which catalyzes the reduction of O_2_ to H_2_O_2_, and lactate oxidase, which forms hydrogen peroxide by oxidizing lactate [44]. Conversely, the presence of *S. thermophilus* in yogurt may have acted to reduce the hydrogen peroxide content. This strain possesses NADH peroxidase, which converts hydrogen peroxide to water while regenerating NAD^+^ [43]. However, even with this protective mechanism, hydrogen peroxide levels in yogurt remain relatively high, potentially affecting the stability of anthocyanins in the smoothie.

Among the fruit semiproducts, strawberry puree showed the highest content of H_2_O_2_, while blueberry and apple products had the lowest. The high content of hydrogen peroxide levels in strawberry puree may have resulted from its accumulation during fruit storage. Vicente et al. (2006) [45] reported that storing strawberries at 20 °C for 1 day led to a 2.5-fold increase in hydrogen peroxide levels compared to freshly harvested fruit. The high hydrogen peroxide content in strawberry puree could also be linked to its high vitamin C content. Model studies have shown that the rate of hydrogen peroxide generation results from the oxidation of ascorbic acid in the presence of oxygen and copper at concentrations relevant to food products [46]. Overall, the interplay between lactic acid bacteria, fruit components, and storage conditions highlights the complexity of maintaining anthocyanin stability in food formulations. For the development of strategies to improve anthocyanin retention, future research could focus on methods to modulate hydrogen peroxide levels in yogurt and fruit semiproducts. Options might include employing antioxidant enzymes or optimizing storage conditions to minimize ROS production. Understanding the impact of hydrogen peroxide on other bioactive compounds and the overall nutritional quality of the smoothie could also offer valuable insights for the development of healthier and more stable yogurt-based products.

### 3.2. Changes in the Counts of Lactic Acid Bacteria during Storage of Smoothies

The changes in the viable cell numbers of *L. delbrueckii* subsp. *bulgaricus* and *S. thermophilus* during the storage of smoothies are presented in Table 3. Initially, the counts of *L. delbrueckii* subsp. *bulgaricus* ranged from 4.4 to 5.4 log CFU/g in strawberry and blueberry smoothies with the addition of 10% and 30% yogurt, respectively. In contrast, the initial population of *S. thermophilus* was approximately 2.7 logarithmic cycles higher, ranging from 7.2 to 8.0 log CFU/g in raspberry and strawberry smoothies containing 10% and 30% yogurt, respectively. These differences could potentially be attributed to the lower number of *L. delbrueckii* subsp. *bulgaricus* cells in the yogurt used for smoothie production (Table S2). Similarly, Vinderola et al. (2000) [47] observed about a 1 log order difference between the counts of *S. thermophilus* and *L. delbrueckii* subsp. *bulgaricus* in yogurt produced with commercial starter cultures. Understanding variations in viable cell numbers and bacterial dynamics is crucial for the production of yogurt-based smoothies, as these factors can impact product consistency, flavor, and potential health benefits. Further investigations into the factors that affect the growth and viability of *S. thermophilus* and *L. delbrueckii* subsp. *bulgaricus* in both yogurt and smoothies could offer valuable insights for optimizing product formulation and storage conditions. Additionally, monitoring changes in viable cell numbers during smoothie storage may assist manufacturers in ensuring product stability throughout its shelf life.

The storage period exerted a significant effect on bacterial numbers in smoothies containing yogurt. A natural decline in bacterial populations during storage is common in fermented products. After 4 weeks of refrigerated storage, the population of *L. delbrueckii* subsp. *bulgaricus* decreased by approximately one logarithmic order, with final counts ranging from 2.3 to 4.5 log CFU/g. *S. thermophilus* exhibited similar behavior during smoothie storage, displaying a decline in cell counts ranging from 0.1 to 2 log orders across all beverages. Notably, there was no significant decline in the number of *S. thermophilus* cells in blueberry smoothies containing 20% and 30% yogurt during the storage period.

According to previous studies, the main factors affecting the viability of lactic acid bacteria during storage include pH, bacterial strain, storage temperature, the presence of hydrogen peroxide and dissolved oxygen, as well as the concentration of inhibitors and nutrients, and the osmotic pressure of the products [35,36,47]. As long as the final viable cell counts stay within acceptable limits and the product maintains its desired sensory and nutritional qualities, this should not raise concerns. Nonetheless, a deeper understanding of the factors influencing bacterial viability can aid manufacturers in designing more stable and consistent products for consumers.

In general, at the end of the storage period, the lowest number of bacterial cells was observed in smoothie samples containing raspberry, while the highest was in beverages with blueberry puree, regardless of the yogurt dosage. The observed difference is likely due to variations in the pH of the fruit purees used in smoothie production, which is associated with differing citric acid content in raspberry and blueberry purees (Table 2). Shah (2000) [36] reported that one of the main factors affecting the survival of lactic acid bacteria is pH and a rapid decline in their numbers in yogurt has been attributed to the accumulation of organic acids. Kailasapthy et al. (2007) [35] also observed that a decreasing pH, caused by the addition of fruit preparations to yogurt, influenced the variability in lactic acid bacteria.

Substances such as polyphenolic and ellagitannin compounds in raspberry puree may also be responsible for the poor survival of yogurt bacteria [48]. Moreover, the low content of ascorbic acid in blueberry puree might enhance the viability of *S. thermophilus*, as observed during smoothie storage. Dave and Shah (1997) [42] reported that counts of *S. thermophilus* were higher when the concentration of ascorbic acid in yogurt decreased. In strawberry and raspberry smoothies, ascorbic acid could lower the redox potential by scavenging oxygen, thus affecting the amount of oxygen required for the activities of *S. thermophilus* [36]. Further research could explore the specific mechanisms by which identified bioactive compounds in raspberry and blueberry purees affect the survival of lactic acid bacteria.

### 3.3. Anthocyanin Composition of Fruit Smoothie

The total anthocyanin contents of strawberry, raspberry, and blueberry smoothies, evaluated using the HPLC method, are presented in Table 4, Table 5 and Table 6. There was a significant variation in the concentration of anthocyanin compounds among different smoothies, with blueberry smoothies exhibiting the highest total anthocyanin content among the tested beverages.

The chromatographic conditions used allowed for the separation and identification of four major anthocyanins in the strawberry smoothie, as well as the detection of other unidentified anthocyanins at much lower concentrations (Figure S1). The primary anthocyanin in the strawberry smoothie was pelargonidin-3-O-glucoside, making up 82% of the total anthocyanins. It was followed, in decreasing order, by pelargonidin-3-malonyl-glucoside, cyanidin-3-O-glucoside, and pelargonidin-3-O-rutinoside. Compared to the smoothie, the fresh strawberries contained relatively higher levels of pelargonidin-3-O-glucoside. Goiffon et al. (1999) [49] showed that pelargonidin-3-O-glucoside accounted for about 91% of the total anthocyanins in strawberries (cv. Elsanta) grown in France. The observed variation could be related to factors such as the cultivar, geographical origin of the fruit, degree of maturity, harvest time, and duration of cold storage [50,51]. Furthermore, the difference in the anthocyanin profile between the fresh fruit and the smoothie in our study might be attributed to the fact that the activities of native oxidative enzymes could affect anthocyanin composition during the production of strawberry puree. According to Oey et al. (2008) [52], β-glucosidase enzymes are responsible for the higher degradation of pelargonidin-3-O-glucoside compared to pelargonidin-3-O-rutinoside in strawberry products. In our study, enzymatic degradation of glucosides could have occurred during the production of strawberry puree, particularly during the “throwing step”.

Cyanidin-3-O-sophoroside was the anthocyanin compound found in the highest concentration in raspberry smoothies, as indicated in Table 5. The second most abundant anthocyanin was cyanidin-3-O-glucoside, accounting for 22% of the total anthocyanin concentration. Small amounts of pelargonidin-3-O-glucosylrutinoside and cyanidin-3-O-glucosylrutinoside were also present. The anthocyanin profile for raspberry yogurts was similar to raspberry juice as reported by Borges et al. (2007) [53]. In the present study, the concentrations of all anthocyanins were determined except for cyanidin-3,5-diglucoside, pelargonidin-3-O-sophoroside, cyanidin-3-O-xylosylrutinoside, and pelargonidin-3-O-glucoside, which were present in the smoothie but were not quantified due to their very low concentrations (Figure S2).

In blueberry smoothies, fourteen monomeric anthocyanins were identified by comparing their retention time and UV–vis spectral characteristics to published values or commercial standards (Figure S3). It was not possible to determine the structure of acylated anthocyanins, so these were not identified. For simplicity, the results are presented for delphinidin, petunidin, and malvidin derivatives, while cyanidin and peonidin derivatives are grouped as their content is relatively minor (Table 6). Malvidin glycosides were the anthocyanin compounds found in the highest concentration in blueberry fruit. The second most abundant anthocyanidin was delphinidin, contributing to 19% of the total anthocyanin concentration. The findings of this study align with those obtained by Li et al. (2016) [54] for the same cultivar grown in China, indicating that malvidin derivatives were the major anthocyanins found in the blueberry.

Immediately after production, a higher concentration of anthocyanins was observed in the control smoothie compared to the smoothie with the addition of yogurt. These differences were statistically significant only for malvidin derivatives in the blueberry smoothie, although similar trends were observed for other anthocyanins. One possible explanation for this effect is that anthocyanins may interact with the proteins present in yogurt, thereby affecting the extraction efficiency of anthocyanins. The predominance of this effect in malvidin derivatives may be due to differences in their substitution patterns on the B-ring. Specifically, malvidin derivatives have more methoxy groups compared to cyanidin and pelargonidin derivatives, which might contribute to a higher degree of interaction with proteins [55]. He et al. (2016) [56] reported that casein proteins interacted with malvidin-3-O-glucoside from grape skins through hydrogen bonding and hydrophobic reactions to form complexes, thereby improving the stability of anthocyanins.

### 3.4. Differences in Anthocyanin Stability in Fruit Smoothies Depending on the Raw Material Used

The content of anthocyanins in the smoothies decreased over a 4-week storage period at 4 °C. The rate of this decrease depended on the type of semiproduct used: for smoothies without yogurt, the decrease was 14%, 9%, and 3% for strawberry, raspberry, and blueberry products, respectively (Table 4, Table 5 and Table 6). The variation in anthocyanin losses across the different fruit-based smoothies could be attributed to both the chemical structure of the anthocyanins and their inherent stability, as well as to other ingredients present in the puree. Strawberry smoothies contained relatively high amounts of vitamin C (Table 2), which might have accelerated the degradation of anthocyanins. Tiwari et al. (2009) [57] observed a strong correlation between the loss of ascorbic acid and pelargonidin-3-glucose during the storage of strawberry juices. They suggested that the ascorbic acid induced degradation of anthocyanins is likely due to the formation of hydrogen peroxide during its oxidation. Similarly, Yuan and Chen (1998) [58] found that ascorbic acid converts to furfural when heated, particularly at low pH levels and under anaerobic conditions. Furfural formation may have occurred during the pasteurization of the strawberry puree, potentially accelerating the degradation of anthocyanins during storage. During production, the homogenization process was carried out under reduced pressure, and the jars were filled with strawberry puree at high temperatures. These steps could limit the amount of oxygen in the product and thus increase the furfural content. Model studies on grape juice have shown that the presence of ascorbic acid significantly accelerates the degradation of anthocyanins from the 11th day of storage at room temperature. This is believed to be linked to a condensation reaction between furfural and the hydroxyl groups of the anthocyanin B ring [59].

Blueberry puree exhibited the highest content of total phenolic compounds among the tested semifinished products, potentially contributing to the high stability of anthocyanins in the blueberry smoothie. In a model experiment, Eiro and Heinonen (2002) [60] investigated the stability of intermolecular copigmentation complexes of anthocyanins with phenolic acids. They found that malvidin-3-glucoside exhibited greater copigmentation effects compared to other anthocyanins. Given that the blueberry smoothie contained significant amounts of malvidin derivatives, such copigmentation between phenolic compounds and anthocyanins might have occurred, thereby protecting the anthocyanins from degradation during storage. Conversely, research by Pangestu et al. (2020) [61] indicated that adding ferulic acid to elderberry juice and purple carrot extract reduced the content of monomeric anthocyanins and the formation of polymeric pigment (pyranoanthocyanins) during storage.

The observed differences in anthocyanin losses during storage among strawberry, raspberry, and blueberry smoothies could stem from differences in the structure and stability of their major anthocyanins. The main anthocyanin in the strawberry smoothie was monoglucoside (pelargonidin-3-O-glucoside), while in the raspberry smoothie, it was cyanidin-3-O-sophoroside, which exhibited higher stability. Previous studies have also confirmed that monoglycosidic substitutions are less stable than diglycosidic substitutions during the storage of strawberry and raspberry juices [62,63]. Strawberry and raspberry smoothies primarily contained cyanidin and pelargonidin derivatives, while the blueberry smoothie featured malvidin derivatives. The stability of anthocyanins increases with the addition of methylation groups to the B ring of anthocyanidin; therefore, malvidin is the most stable among the anthocyanidins, a finding corroborated by our research. Similarly, Reque et al. (2014) [64] found that delphinidin and cyanidin glucosides were the least stable anthocyanins during the storage of blueberry juice, while malvidin and peonidin derivatives proved to be the most stable. This contrasts with a study by Antonio-Gomez et al. (2023) [65], which demonstrated that cyanidin-3-O-galactoside was the most stable during the storage of isotonic beverages containing Chagalapoli anthocyanins, including malvidin and petunidin derivatives. The blueberry smoothie also contained acylated anthocyanins, known for their good stability. According to research by Enaru et al. (2021) [66], acylation plays a crucial role in enhancing the stability of anthocyanins. On the other hand, some model systems have shown that increased acylation does not necessarily improve stability during the storage of purified anthocyanins [67]. This suggests that, in addition to the anthocyanin structure, other environmental factors may accelerate anthocyanin degradation.

### 3.5. Effect of Yogurt Addition on Anthocyanin Stability during Cold Storage of Smoothie

In all smoothies after 4 weeks of storage, the addition of 30% yogurt led to significantly greater total anthocyanin losses compared to those without yogurt (as shown in Table 4, Table 5 and Table 6). When yogurt was added at a level of 20%, differences in anthocyanin losses were observed only for strawberry and raspberry smoothies. Conversely, there were no statistically significant differences in anthocyanin losses between smoothies without yogurt and those with a 10% yogurt addition across all tested products. A comparison of the anthocyanin content in control smoothies (without added yogurt) and those containing 30% yogurt revealed differences of 1.84, 0.98, and 0.88 mg/100 g for strawberry, raspberry, and blueberry products, respectively, after 4 weeks of storage. This suggests that the 30% yogurt addition negatively impacts anthocyanin stability, but the extent of this impact varies depending on the fruit used in production. These variations could be due to the differing chemical structures of the anthocyanins in these fruits, the composition of the beverages, or variations in the survival rates of lactic acid bacteria.

Differences in anthocyanin losses during storage between control smoothies and those with 30% and 20% yogurt (in the cases of strawberry and raspberry) could be attributed to several factors. The first factor might be the increase in smoothie pH due to the addition of yogurt. For products with 30% yogurt, the pH levels were 3.67, 3.49, and 3.92 for strawberry, raspberry, and blueberry smoothies, respectively (Table S1). The average pH difference between smoothies without yogurt and those with a 30% yogurt addition was 0.25. Moldovan et al. (2012) [68] indicated a significant impact of pH on anthocyanin stability in cranberry fruits during storage. In comparing half-lives, anthocyanins at a pH of 3 were 4.7 times more stable than those at a pH of 7. However, these tested pH ranges are quite broad and do not necessarily reflect the pH levels found in smoothie products. Therefore, it is important to conduct model studies to confirm or reject the hypothesis that a decrease in pH—caused by the addition of yogurt—affects changes in anthocyanin levels in smoothies stored at refrigerated temperatures.

A very important factor that could have influenced the higher degradation of anthocyanins observed during storage after yogurt was added is the relatively high concentration of hydrogen peroxide in this semifinished product (Table 2). Studies evaluating the effect of hydrogen peroxide on anthocyanins confirm that it accelerates the degradation of anthocyanins. This acceleration increases with the dosage of hydrogen peroxide and the temperature at which the storage occurs [10]. Various anthocyanins have also been shown to exhibit different sensitivities to H_2_O_2_. For example, strawberry anthocyanins degrade much more quickly in the presence of H_2_O_2_ compared to those found in cherries and pomegranates. This is thought to be related both to the different anthocyanin compositions and to variations in the levels of other polyphenolic compounds. This is relevant because hydrogen peroxide can catalyze the oxidation of other phenolic compounds into quinones, which can, in turn, contribute to the degradation of anthocyanins. Studies evaluating the effect of hydrogen peroxide on anthocyanin stability have mainly focused on its use in aseptic juice packaging and as a disinfectant. Consequently, the concentrations tested in those studies significantly exceed the amounts of hydrogen peroxide likely to be introduced into beverages through the addition of yogurt [10,69]. Therefore, a study using a model system that adjusts hydrogen peroxide levels to those found in yogurt would seem to be justified.

When comparing the observed viability of lactic acid bacteria (Table 3) to anthocyanin losses during smoothie storage, it is noteworthy that the blueberry product, which showed no significant decrease in the amount of *S. thermophilus* cells, also demonstrated high anthocyanin stability. This could be related to this particular strain’s ability to reduce hydrogen peroxide levels [43]. Conversely, in the raspberry smoothie, a significant drop in cell counts was observed during storage. However, this decrease did not correspond to a higher loss of anthocyanins, suggesting that other factors may also have influenced the outcome. The presence of *S. thermophilus*, equipped with NADH peroxidase activity that reduces hydrogen peroxide to water, could potentially mitigate the oxidative degradation of anthocyanins. This might result in higher anthocyanin stability in the smoothie. To develop a comprehensive understanding of the mechanisms affecting anthocyanin stability in smoothies, future research could focus on examining the interactions between these compounds and lactic acid bacteria. This information could be useful for optimizing both product formulations and storage practices to improve anthocyanin retention in yogurt-based smoothies.

An important factor that may have accelerated the degradation of anthocyanins in smoothies with added yogurt is the enzymatic activity of the lactic acid bacteria used in its production. Recent studies suggest that lactic acid bacteria can produce β-glucosidase, an enzyme capable of hydrolyzing the glycosidic bonds of anthocyanins to produce anthocyanidins, thereby altering their profile in the product [11]. In research by Braga et al. (2018) [70] on the enzymatic activity of *Lactobacillus* and *Bifidobacterium* strains, it was found that the strains evaluated did produce β-glucosidase enzymes. These strains also could alter the chromatographic profile of the primary anthocyanin found in jussara pulp. In our chromatographic analysis of anthocyanins in stored smoothies, we did not observe the appearance of anthocyanidins on the chromatograms. This absence could be attributed to the fact that these forms are highly unstable and prone to rapid degradation.

Another key aspect related to the varying stability of anthocyanins in smoothies after the addition of yogurt concerns changes in nutrient content, which can either positively or negatively influence anthocyanin stability. The yogurt used in production had a protein content of 3.26% (Table S2), as determined by the Kjeldahl method; therefore, its addition altered the protein composition of the resultant products. Recent research indicates that products containing both anthocyanins and proteins exhibit hydrophobic, electrostatic, and hydrogen bonding interactions between the two, forming a more stable structure as a result. The protective effect of proteins on anthocyanins varies in stored products and is dependent on both the storage conditions and the specific structure of the anthocyanin involved [13]. For example, Chung et al. (2017) [14] found that amino acids and peptides could also enhance the stability of anthocyanins derived from purple carrots. In this study, the formation of anthocyanin–protein complexes could have been limited by several factors: low storage temperature, short storage time, and the relatively small amount of protein present in the smoothies.

The incorporation of yogurt into the smoothie also led to an increase in fat content, which stood at 3.2% in the yogurt (Table S2). Wallace and Giusti (2008) [15] demonstrated in their studies on anthocyanin stability during yogurt storage with varying fat levels (0–4%) that acylated anthocyanins from purple carrot extracts displayed increased stability in the presence of higher fat content. Furthermore, adding yogurt altered the sugar composition of these products. Specifically, the more yogurt added, the higher the lactose content and the lower the levels of sucrose and fructose originating from the fruit puree and apple juice (Table 2). Notably, fructose and lactose have a greater impact on anthocyanin degradation than glucose and sucrose do [71].

Another factor that could influence the stability of anthocyanins in the smoothie may not only be the increased addition of yogurt but also the reduced proportion of juice and apple puree. These components may have contained anthocyanin-stabilizing ingredients. For instance, Buchweitz et al. (2013) [72] showed in model studies that adding apple pectin to strawberry anthocyanin extract increased the stability of the pigments during storage at 20 °C. The apple semifinished products used in the smoothie production were rich in phenolic compounds (Table 2). Numerous studies have demonstrated that flavonoids serve as effective copigments, enhancing pigment stability by protecting anthocyanin chromophores from nucleophilic attacks [73]. Moreover, the apple juice and puree contained significant amounts of malic acid. Huang et al. (2023) [74] revealed that the addition of malic acid to roselle extract improves the thermostability of anthocyanins.

### 3.6. Role of pH, H_2_O_2_ and Addition of Cell-Free Extracts on Stability of Anthocyanins in the Model System

A model experiment was used to assess the effect of pH, hydrogen peroxide, and the addition of cell-free extracts on the stability of anthocyanins isolated from strawberries, raspberries, and blueberries. The experimental conditions were tailored to match the parameters of previously analyzed fruit smoothies. The range of pH considered (3.0–4.5) corresponded to that of the fruit puree, juice, and yogurt. The concentrations of hydrogen peroxide investigated also aligned with those found in fruit semiproducts and yogurt (1, 3, and 6 µg/mL). *S. thermophilus* and *L. delbrueckii* subsp. *bulgaricus*, which is used in yogurt production, served as monocultures in this model experiment.

**Figure 1:**
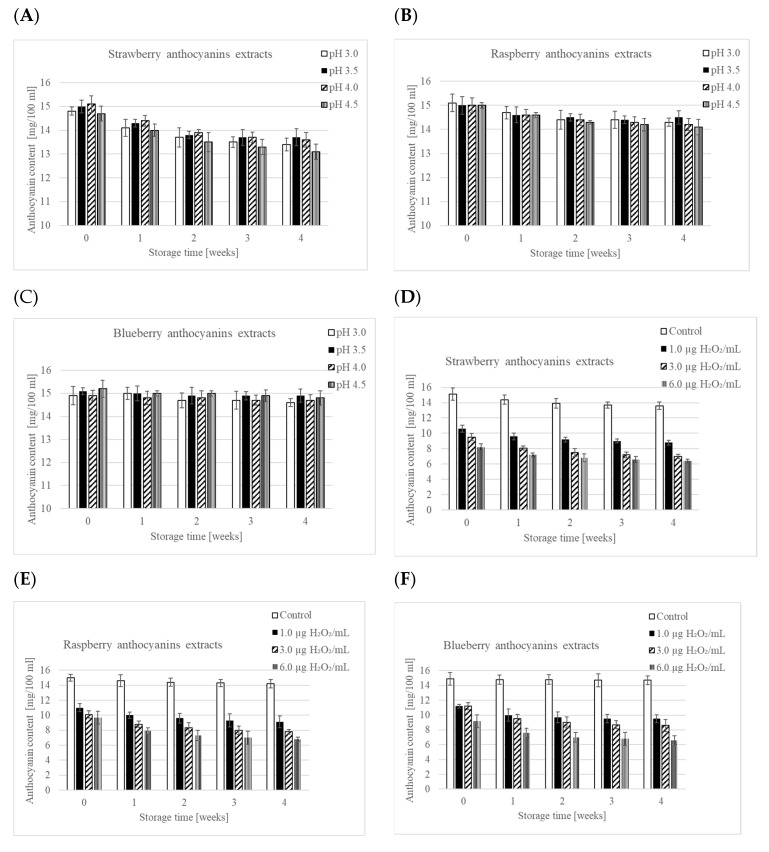

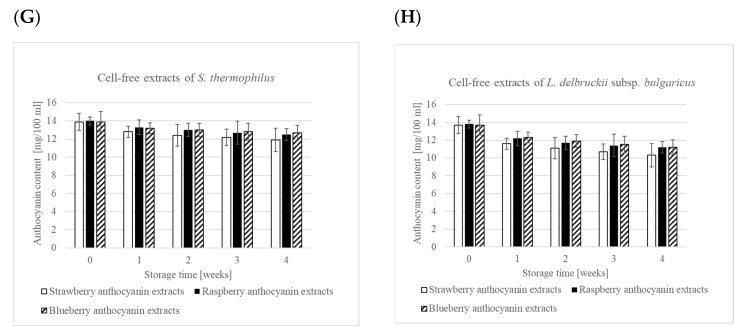
Changes in total anthocyanin content (mg/100 mL) during storage of purified pigment extracts from strawberry, raspberry, and blueberry fruits in different conditions: pH (**A**–**C**), H_2_O_2_ concentration (**D**–**F**), and cell-free extract presence (**G**–**H**).

During storage of the anthocyanin extracts at varying pH levels (3, 3.5, 4, and 4.5), there was a gradual decline in anthocyanin content (Figure 1A–C). Similar to what was observed in the smoothies, the most significant drop in anthocyanins occurred in extracts derived from strawberries, while the least occurred in those from blueberries. Over a 4-week storage period, the anthocyanin content decreased on average by 10%, 5%, and 2% for strawberries, raspberries, and blueberries, respectively. These anthocyanin losses during storage in the model study were lower compared to those in the smoothie without yogurt. This discrepancy may arise from the presence of other components such as sugars, vitamins, pectin, organic acids, and polyphenolic compounds other than anthocyanins in the smoothie, from which the tested extracts were purified. In addition, the fruit puree’s production involved thermal processing, which could have also contributed to the observed differences in anthocyanin stability between smoothies and extracts. Interestingly, the study found that pH levels in the range of 3–4.5 did not significantly influence the observed losses in anthocyanins during storage of the extracts. Luna-Vital et al. (2017) [7] reported similar findings, showing that the storage of purple corn extracts at pH levels of 3.0, 3.5, and 4.0 did not significantly affect the observed changes in total anthocyanin content when measured using the spectrophotometric method. Moreover, these studies used a much higher storage temperature (22 °C) and a longer storage time (12 weeks).

Changes in anthocyanin content during storage of extracts after adding hydrogen peroxide in amounts of 1.0, 3.0, and 6.0 µg/mL are illustrated in Figure 1D–F. A significant decrease in anthocyanin content was observed immediately following the addition of hydrogen peroxide. This is consistent with a study by Alexandre et al. (2012) [75] which reported a 38% immediate decrease in anthocyanin content when strawberry fruit was immersed in a 5% H_2_O_2_ disinfectant solution. An increase in the concentration of hydrogen peroxide correspondingly accelerated the degradation of anthocyanins, echoing findings by Ruenroengklin et al. (2009) [76] on litchi anthocyanins. However, the decline in anthocyanin content was not directly proportional to the concentration of hydrogen peroxide added. For example, the initial difference in anthocyanin content for extracts with 3.0 and 6.0 µg H_2_O_2_/mL was 1.3, 0.4, and 2.0 mg/100 mL for strawberry, raspberry, and blueberry, respectively. Storing the extracts with added peroxide led to a gradual decrease in anthocyanin levels, but this decline was not as dramatic as the one observed immediately after the addition of hydrogen peroxide (Figure S3). Interestingly, the variations in anthocyanin stability across different fruits became less evident when hydrogen peroxide was introduced. After 4 weeks of storage, anthocyanin contents in extracts with added hydrogen peroxide were 6.4, 6.8, and 6.6 mg/100 mL for strawberry, raspberry, and blueberry, respectively. This contrasts with findings for sour cherry nectar, pomegranate, and strawberry juices, where differing sensitivities to hydrogen peroxide were noted [10]. However, it is important to mention that those studies used higher concentrations of hydrogen peroxide and stored the beverages at higher temperatures than the extracts in this study, which could account for the divergent outcomes observed.

In the most recent model experiment, cell-free extracts of *S. thermophilus* and *L. delbrueckii* subsp. *bulgaricus* were introduced to anthocyanin extracts. The ensuing pigment changes are depicted in Figure 1G,H. Upon the addition of these cell-free extracts, an immediate decrease in anthocyanins was noted, averaging 7.1% for *S. thermophilus* and 8.4% for *L. delbrueckii* subsp. *bulgaricus*, across all tested extracts. This significant drop in anthocyanins mirrors the decrease observed in a similar model experiment involving the addition of hydrogen peroxide. This observation aligns with research by Sasaki et al. (2014) [9], who showed that *L. delbrueckii* subsp. *bulgaricus* produces hydrogen peroxide during milk fermentation, and *S. thermophilus* does not generate detectable levels of the same under identical conditions. They also suggested that the medium itself might have the ability to reduce the amount of hydrogen peroxide produced. Subsequent studies could delve deeper into the enzymatic activities of lactic acid bacteria and their effects on anthocyanins. Such research could include identifying and characterizing the specific enzymes behind anthocyanin degradation, as well as investigating how the smoothie’s overall composition and environmental conditions influence enzyme activity. Gaining this understanding would be pivotal in crafting strategies to preserve anthocyanins and other bioactive compounds in yogurt-based smoothies, thereby optimizing their potential health benefits and overall product quality.

Storing the obtained extracts under refrigerated conditions led to a further decline in anthocyanin levels. After a 4-week storage period, the average losses were 18% for *S. thermophilus* and 27% for *L. delbrueckii* subsp. *bulgaricus* (Figure S2). Information about the effect of microorganisms on anthocyanin stability is scant and published results to date are inconsistent. For instance, Ricci et al. (2018) [77] demonstrated that zero-growth strains of *Lactobacillus casei* could enhance the anthocyanin content in elderberry juice, specifically cyanidin-3-O-sambubioside and cyanidin-3-O-glucoside. Conversely, Wu et al. (2021) [55] found that the most significant decrease in anthocyanin content in both blackberry and blueberry juices over a 48-h fermentation period occurred when *S. thermophilus* was used. Moreover, Ávila et al. (2009) [78] reported that strains of lactic acid bacteria exhibit β-glucosidase activity and can degrade compounds like malvidin-3-O-glucoside and delphinidin-3-O-glucoside to varying extents, likely through glycosidic bond cleavage. Acar and Yüksekdağ (2023) [12] compared the β-glucosidase activity of different lactic acid bacterial strains and found that it was influenced not only by the strain but also by the environmental pH and temperature. They identified an optimum condition of pH 7.5 and a temperature of 30 °C—conditions quite different from the storage conditions for the smoothies and extracts in this study. In addition, Pham et al. (2000) [79] revealed that the highest β-glucosidase activity was in the cell-bound fraction, suggesting that the enzyme primarily resides on the cell surface rather than intracellularly. This could have implications for the stability of anthocyanins in experiments using cell-free extracts. Finally, Michlmayr et al. (2010) [80] reported that the cell-surface localization of β-glucosidase varies depending on the lactic acid bacterial species.

## 4. Conclusions

In summary, our research has provided valuable insights into the impact of the addition of yogurt on the stability of anthocyanins during a 4-week refrigerated storage period in fruit smoothies. We observed a notable adverse effect, particularly when yogurt constituted 30% of all tested beverages, and at 20% in the case of strawberry and raspberry smoothies. The degree of impact varied among the beverages studied, with strawberry smoothies exhibiting the most pronounced effect and the blueberry variant the least. Moreover, our study revealed the substantial influence of the addition of yogurt on anthocyanin stability in fruit smoothies. The presence of *S. thermophilus*, with high viability noted in stored blueberry smoothies, may have contributed to anthocyanin stability through the reduction of hydrogen peroxide concentrations. Additionally, model experiments conducted on purified anthocyanin extracts demonstrated that the pH range of 3.0–4.5 did not significantly affect anthocyanin stability during refrigerated storage. Conversely, significant effects were observed with the addition of hydrogen peroxide and cell-free extracts, particularly those derived from *L. delbrueckii* subsp. *bulgaricus*. In conclusion, our study sheds light on the challenges posed by the addition of yogurt to anthocyanin stability in fruit smoothies under refrigerated conditions. To gain a more comprehensive understanding of the underlying mechanisms and to formulate effective preservation strategies, future research should delve into the intricate interactions between lactic acid bacteria, anthocyanins, and their microenvironment. These insights could have practical implications for the food industry, potentially paving the way for the development of healthier and more stable yogurt-based products.

## Figures and Tables

**Table 1:** Recipe ingredients for the preparation of 1 kg of smoothie.

Semiproducts	Strawberry Smoothie				Raspberry Smoothie				Blueberry Smoothie			
yogurt	0	100 g	200 g	300 g	0	100 g	200 g	300 g	0	100 g	200 g	300 g
strawberry puree	400 g	400 g	400 g	400 g	-	-	-	-	-	-	-	-
raspberry puree	-	-	-	-	400 g	400 g	400 g	400 g	-	-	-	-
blueberry puree	-	-	-	-	-	-	-	-	400 g	400 g	400 g	400 g
apple puree	300 g	250 g	200 g	150 g	300 g	250 g	200 g	150 g	300 g	250 g	200 g	150 g
apple juice	300 g	250 g	200 g	150 g	300 g	250 g	200 g	150 g	300 g	250 g	200 g	150 g

**Table 2:** Physicochemical parameters of semiproducts used in the production of smoothies.

Parameters	Strawberry Puree	Raspberry Puree	Blueberry Puree	Apple Puree	Apple Juice	Yogurt
Soluble solids (°Bix)	9.1 ^D^ ± 0.1	11.6 ^B^ ± 0.4	12.8 ^A^ ± 0.6	11.3 ^B^ ± 0.2	10.4 ^C^ ± 0.5	nt
Fructose (g/100 g)	3.3 ^D^ ± 0.2	4.0 ^C^ ± 0.1	5.5 ^B^ ± 0.3	6.1 ^A^ ± 0.1	5.8 ^B^ ± 0.0	nd
Glucose (g/100 g)	3.1 ^B^ ± 0.3	3.5 ^B^ ± 0.5	5.1 ^A^ ± 0.5	1.5 ^C^ ± 0.2	1.5 ^C^ ± 0.1	0.02 ^D^ ± 0.0
Saccharose (g/100 g)	0.6 ^C^ ± 0.1	1.4 ^B^ ± 0.2	nd	2.2 ^A^ ± 0.4	2.5 ^A^ ± 0.3	nd
Lactose (g/100 g)	nd	nd	nd	nd	nd	3.8
Galactose (g/100 g)	nd	nd	nd	nd	nd	1.7
Titratable acidy (g/100 g) *	0.80 ^B^ ± 0.02	1.20 ^A^ ± 0.04	0.45 ^D^ ± 0.04	0.50 ^CD^ ± 0.01	0.54 ^C^ ± 0.04	0.76 ^B^ ± 0.04
pH	3.38 ^D^ ± 0.05	3.19 ^E^ ± 0.08	3.61 ^B^ ± 0.09	3.44 ^C^ ± 0.04	3.45 ^C^ ± 0.03	4.17 ^A^ ± 0.04
Citric acid (g/100 g)	0.62 ^B^ ± 0.03	1.08 ^A^ ± 0.10	0.41 ^C^ ± 0.07	0.04 ^E^ ± 0.00	0.04 ^E^ ± 0.00	0.11 ^D^ ± 0.02
Malic acid (g/100 g)	0.12 ^B^ ± 0.03	0.10 ^B^ ± 0.02	0.02 ^C^ ± 0.00	0.47 ^A^ ± 0.07	0.45 ^A^ ± 0.04	nd
Lactic acid (g/100 g)	nd	nd	nd	nd	nd	0.62
Vitamin C (mg/100 g)	22.6 ^A^ ± 1.4	10.8 ^B^ ± 0.9	2.8 ^C^ ± 0.2	2.7 ^C^ ± 0.3	2.2 ^D^ ± 0.2	0.3 ^E^ ± 0.0
Phenolic content (mg/100 g)	235.7 ^C^ ± 7.7	289.1 ^B^ ± 9.0	338.6 ^A^ ± 12.2	167.7 ^D^ ± 6.8	124.0 ^E^ ± 2.1	5.6 ^F^ ± 0.8
Hydrogen peroxide content (µg/g)	2.4 ^B^ ± 0.3	1.8 ^B^ ± 0.3	1.1 ^C^ ± 0.2	1.2 ^C^ ± 0.1	1.2 ^C^ ± 0.2	5.90 ^A^ ± 0.8

nd—not detected; nt—not tested; ± standard deviation * g of citric acid per 100 g for strawberry, raspberry and blueberry purees; g of malic acid per 100 g of apple puree and juice and g of lactic acid per 100 g of yogurt. ^A–F^—means in the same line followed by different uppercase represents significant difference.

**Table 3:** Changes in the population of lactic acid bacteria (log CFU/g) during cold storage of smoothies.

Type of Smoothie	Addition of Yogurt	*L. delbrueckii* subsp. *bulgaricus*					*S. thermophilus*				
		Storage Time (Week)					Storage Time (Week)				
		0	1	2	3	4	0	1	2	3	4
Strawberry smoothie	10%	4.4 ^Ab^ ± 0.1	4.2 ^Bb^ ± 0.1	4.1 ^BCb^ ± 0.0	4.1 ^BCb^ ± 0.1	4.0 ^Cb^ ± 0.1	7.4 ^Ab^ ± 0.1	7.3 ^Ab^ ± 0.1	6.9 ^Bb^ ± 0.1	6.5 ^Cb^ ± 0.1	5.9 ^Db^ ± 0.2
	20%	4.6 ^Aab^ ± 0.2	4.6 ^Aa^ ± 0.1	4.5 ^Aab^ ± 0.1	4.5 ^Aab^ ± 0.0	4.3 ^Bab^ ± 0.1	7.8 ^Aa^ ± 0.1	7.4 ^Bb^ ± 0.0	7.3 ^Bab^ ± 0.1	6.8 ^Cab^ ± 0.2	6.1 ^Dab^ ± 0.1
	30%	4.9 ^Aa^ ± 0.2	4.7 ^ABa^ ± 0.1	4.7 ^ABa^ ± 0.1	4.9 ^Aa^ ± 0.1	4.5 ^Ba^ ± 0.2	8.0 ^Aa^ ± 0.2	7.7 ^Ba^ ± 0.1	7.4 ^Ca^ ± 0.1	7.0 ^Da^ ± 0.1	6.4 ^Ea^ ± 0.2
Raspberry smoothie	10%	4.6 ^Ab^ ± 0.2	3.6 ^Bb^ ± 0.1	3.1 ^Cb^ ± 0.2	2.4 ^Db^ ± 0.1	2.3 ^Db^ ± 0.1	7.2 ^Ab^ ± 0.2	6.4 ^Bb^ ± 0.1	6.1 ^Cb^ ± 0.1	5.8 ^Db^ ± 0.1	5.5 ^Eb^ ± 0.2
	20%	5.0 ^Aa^ ± 0.1	3.7 ^Bb^ ± 0.1	3.3 ^Cb^ ± 0.1	2.4 ^Db^ ± 0.0	2.4 ^Dab^ ± 0.1	7.6 ^Aa^ ± 0.1	6.5 ^Bb^ ± 0.2	6.2 ^Cb^ ± 0.1	6.1 ^Cb^ ± 0.1	5.6 ^Db^ ± 0.2
	30%	5.2 ^Aa^ ± 0.2	4.0 ^Ba^ ± 0.1	3.7 ^Ca^ ± 0.1	2.8 ^Da^ ± 0.2	2.7 ^Da^ ± 0.2	7.8 ^Aa^ ± 0.1	6.9 ^Ba^ ± 0.1	6.5 ^Ca^ ± 0.1	6.2 ^Da^ ± 0.1	6.0 ^Da^ ± 0.1
Blueberry smoothie	10%	5.1 ^Ab^ ± 0.1	4.7 ^Bb^ ± 0.1	4.6 ^BCc^ ± 0.1	4.5 ^Cb^ ± 0.1	4.0 ^Db^ ± 0.2	7.5 ^Ab^ ± 0.1	7.2 ^Bc^ ± 0.1	7.2 ^Bc^ ± 0.0	7.2 ^Bc^ ± 0.1	7.1 ^Bc^ ± 0.0
	20%	5.3 ^Aab^ ± 0.0	4.9 ^Bab^ ± 0.2	4.9 ^Bb^ ± 0.1	4.6 ^Cb^ ± 0.1	4.3 ^Dab^ ± 0.1	7.6 ^Ab^ ± 0.1	7.6 ^Ab^ ± 0.0	7.5 ^Ab^ ± 0.1	7.5 ^Ab^ ± 0.1	7.5 ^Ab^ ± 0.1
	30%	5.4 ^Aa^ ± 0.1	5.1 ^Ba^ ± 0.1	5.0 ^Ba^ ± 0.1	4.9 ^Ba^ ± 0.1	4.5 ^Ca^ ± 0.2	7.9 ^Aa^ ± 0.2	7.8 ^Aa^ ± 0.1	7.9 ^Aa^ ± 0.1	7.7 ^Aa^ ± 0.0	7.7 ^Aa^ ± 0.1

± standard deviation. ^A–E^—means in the same line followed by different uppercase represents significant difference individually for each strain. ^a–c^—means in the same column followed by different lowercase represents significant difference individually for each fruit.

**Table 4:** Changes of anthocyanin content (mg/100 g) during storage of strawberry smoothie depending on the addition of yogurt.

Anthocyanins	Addition of Yogurt	Storage Time (Week)				
		0	1	2	3	4
cyanidin-3-O-glucoside	0%	0.51 ^Aa^ ± 0.02	0.48 ^ABa^ ± 0.02	0.45 ^BCa^ ± 0.02	0.43 ^CDa^ ± 0.01	0.41 ^Da^ ± 0.01
	10%	0.49 ^Aa^ ± 0.03	0.46 ^ABab^ ± 0.02	0.44 ^BCab^ ± 0.01	0.44 ^BCa^ ± 0.00	0.42 ^Ca^ ± 0.01
	20%	0.50 ^Aa^ ± 0.03	0.44 ^Bb^ ± 0.02	0.40 ^BCbc^ ± 0.02	0.38 ^CDb^ ± 0.01	0.36 ^Db^ ± 0.02
	30%	0.50 ^Aa^ ± 0.04	0.43 ^Bb^ ± 0.03	0.39 ^BCc^ ± 0.01	0.37 ^CDb^ ± 0.02	0.34 ^Db^ ± 0.02
pelargonidin-3-O-glucoside	0%	7.38 ^Aa^ ± 0.2	6.93 ^Ba^ ± 0.3	6.68 ^BCa^ ± 0.1	6.49 ^Ca^ ± 0.2	6.40 ^Ca^ ± 0.2
	10%	7.31 ^Aa^ ± 0.1	6.70 ^Ba^ ± 0.2	6.38 ^BCa^ ± 0.2	6.08 ^Ca^ ± 0.3	5.99 ^Ca^ ± 0.2
	20%	7.32 ^Aa^ ± 0.4	6.28 ^Bb^ ± 0.2	5.72 ^Cb^ ± 0.2	5.37 ^Db^ ± 0.2	5.15 ^Db^ ± 0.3
	30%	7.30 ^Aa^ ± 0.0	6.01 ^Bb^ ± 0.2	5.58 ^Cb^ ± 0.2	5.10 ^Db^ ± 0.2	4.83 ^Eb^ ± 0.1
pelargonidin-3-O-rutinoside	0%	0.26 ^Aa^ ± 0.02	0.24 ^Aa^ ± 0.02	0.23 ^Aa^ ± 0.02	0.23 ^Aa^ ± 0.01	0.20 ^Aa^ ± 0.02
	10%	0.24 ^Aab^ ± 0.04	0.22 ^Aab^ ± 0.02	0.21 ^Aa^ ± 0.01	0.20 ^Ab^ ± 0.02	0.20 ^Aa^ ± 0.02
	20%	0.22 ^Ab^ ± 0.01	0.21 ^Ab^ ± 0.01	0.21 ^Aa^ ± 0.02	0.20 ^Ab^ ± 0.01	0.20 ^Aa^ ± 0.01
	30%	0.22 ^Ab^ ± 0.02	0.18 ^ABc^ ± 0.02	0.17 ^Bb^ ± 0.01	0.16 ^Bc^ ± 0.02	0.15 ^Bb^ ± 0.02
pelargonidin *3*-malonyl-glucoside	0%	0.92 ^Aa^ ± 0.02	0.88 ^Ba^ ± 0.01	0.86 ^BCa^ ± 0.02	0.85 ^Ca^ ± 0.01	0.83 ^Ca^ ± 0.03
	10%	0.90 ^Aa^ ± 0.01	0.86 ^Ba^ ± 0.02	0.83 ^BCa^ ± 0.01	0.80 ^CDb^ ± 0.02	0.77 ^Dab^ ± 0.03
	20%	0.91 ^Aa^ ± 0.03	0.82 ^Bb^ ± 0.01	0.77 ^Cb^ ± 0.02	0.74 ^CDc^ ± 0.02	0.72 ^Dbc^ ± 0.02
	30%	0.89 ^Aa^ ± 0.03	0.79 ^Bb^ ± 0.02	0.74 ^Cb^ ± 0.01	0.71 ^CDc^ ± 0.02	0.68 ^Dc^ ± 0.02
Total	0%	9.07 ^Aa^ ± 0.2	8.53 ^Ba^ ± 0.2	8.22 ^Ca^ ± 0.1	8.00 ^CDa^ ± 0.3	7.84 ^Da^ ± 0.2
	10%	8.94 ^Aa^ ± 0.4	8.24 ^Ba^ ± 0.1	7.86 ^Ca^ ± 0.2	7.52 ^CDa^ ± 0.2	7.38 ^Da^ ± 0.3
	20%	8.95 ^Aa^ ± 0.4	7.75 ^Bb^ ± 0.3	7.10 ^Cb^ ± 0.2	6.69 ^Db^ ± 0.2	6.43 ^Db^ ± 0.3
	30%	8.91 ^Aa^ ± 0.5	7.41 ^Bb^ ± 0.3	6.88 ^Cb^ ± 0.2	6.34 ^Db^ ± 0.2	6.00 ^Eb^ ± 0.1

± standard deviation. ^A–E^—Same line followed by different uppercase represents a significant difference (*p* ≤ 0.05). ^a–c^—Same column followed by different lowercase represents a significant difference (*p* ≤ 0.05).

**Table 5:** Changes in anthocyanin content (mg/100 g) during storage of raspberry smoothie depending on the addition of yogurt.

Anthocyanins	Addition of Yogurt	Storage Time (Week)				
		0	1	2	3	4
cyanidin-3-O-sophoroside	0%	8.27 ^Aa^ ± 0.1	8.04 ^Ba^ ± 0.1	7.84 ^BCa^ ± 0.2	7.71 ^CDa^ ± 0.1	7.54 ^Da^ ± 0.1
	10%	8.22 ^Aa^ ± 0.1	7.94 ^Bab^ ± 0.1	7.70 ^Cab^ ± 0.1	7.54 ^CDab^ ± 0.2	7.33 ^Dab^ ± 0.1
	20%	8.20 ^Aa^ ± 0.2	7.61 ^Bbc^ ± 0.1	7.45 ^Cbc^ ± 0.0	7.27 ^CDbc^ ± 0.2	7.06 ^Dbc^ ± 0.2
	30%	8.20 ^Aa^ ± 0.3	7.52 ^Bc^ ± 0.2	7.31 ^BCc^ ± 0.2	7.07 ^CDc^ ± 0.1	6.89 ^Dc^ ± 0.2
cyanidin-3-O-glucosylrutinoside	0%	0.12 ^Aa^ ± 0.02	0.12 ^Aa^ ± 0.02	0.12 ^Aa^ ± 0.01	0.11 ^Aa^ ± 0.01	0.11 ^Aa^ ± 0.01
	10%	0.11 ^Aa^ ± 0.02	0.11 ^Aa^ ± 0.00	0.11 ^Aa^ ± 0.00	0.10 ^Aa^ ± 0.02	0.10 ^Aa^ ± 0.02
	20%	0.12 ^Aa^ ± 0.01	0.12 ^Aa^ ± 0.01	0.11 ^Aa^ ± 0.01	0.10 ^Aa^ ± 0.01	0.10 ^Aa^ ± 0.01
	30%	0.12 ^Aa^ ± 0.03	0.11 ^Aa^ ± 0.01	0.11 ^Aa^ ± 0.01	0.10 ^Aa^ ± 0.01	0.10 ^Aa^ ± 0.01
cyanidin-3-O-glucoside	0%	2.47 ^Aa^ ± 0.1	2.40 ^Aa^ ± 0.0	2.38 ^Aa^ ± 0.0	2.33 ^ABa^ ± 0.1	2.27 ^Ba^ ± 0.0
	10%	2.45 ^Aa^ ± 0.1	2.34 ^ABa^ ± 0.0	2.29 ^BCa^ ± 0.0	2.21 ^CDb^ ± 0.0	2.15 ^Db^ ± 0.0
	20%	2.46 ^Aa^ ± 0.1	2.24 ^Bb^ ± 0.0	2.18 ^BCb^ ± 0.0	2.11 ^CDc^ ± 0.0	2.02 ^Dc^ ± 0.1
	30%	2.42 ^Aa^ ± 0.1	2.17 ^Bb^ ± 0.0	2.16 ^Bb^ ± 0.0	2.15 ^Bc^ ± 0.0	2.00 ^Cc^ ± 0.0
cyanidin-3-O-rutinoside	0%	0.25 ^Aa^ ± 0.01	0.24 ^Aa^ ± 0.02	0.24 ^Aa^ ± 0.02	0.23 ^Aa^ ± 0.02	0.23 ^Aa^ ± 0.01
	10%	0.24 ^Aa^ ± 0.01	0.23 ^Aa^ ± 0.02	0.23 ^Aab^ ± 0.01	0.23 ^Aa^ ± 0.01	0.23 ^Aa^ ± 0.01
	20%	0.22 ^Aa^ ± 0.02	0.21 ^ABa^ ± 0.01	0.20 ^ABb^ ± 0.01	0.19 ^ABb^ ± 0.01	0.18 ^Bb^ ± 0.01
	30%	0.22 ^Aa^ ± 0.02	0.22 ^Aa^ ± 0.00	0.21 ^Aab^ ± 0.01	0.20 ^Aab^ ± 0.00	0.19 ^Aab^ ± 0.02
pelargonidin-3-O-glucosylrutinoside	0%	0.14 ^Aa^ ± 0.01	0.13 ^Aa^ ± 0.01	0.13 ^Aa^ ± 0.02	0.13 ^Aa^ ± 0.02	0.12 ^Aa^ ± 0.02
	10%	0.14 ^Aa^ ± 0.02	0.14 ^Aa^ ± 0.02	0.13 ^Aa^ ± 0.01	0.13 ^Aa^ ± 0.00	0.13 ^Aa^ ± 0.01
	20%	0.12 ^Aa^ ± 0.03	0.12 ^Aa^ ± 0.01	0.11 ^Aa^ ± 0.02	0.10 ^Aa^ ± 0.02	0.10 ^Aa^ ± 0.02
	30%	0.14 ^Aa^ ± 0.02	0.13 ^Aa^ ± 0.00	0.13 ^Aa^ ± 0.01	0.12 ^Aa^ ± 0.01	0.11 ^Aa^ ± 0.02
Total	0%	11.25 ^Aa^ ± 0.1	10.93 ^Ba^ ± 0.1	10.71 ^BCa^ ± 0.1	10.51 ^CDa^ ± 0.2	10.27 ^Da^ ± 0.2
	10%	11.16 ^Aa^ ± 0.2	10.76 ^Ba^ ± 0.1	10.46 ^BCab^ ± 0.2	10.21 ^CDab^ ± 0.1	9.94 ^Dab^ ± 0.1
	20%	11.12 ^Aa^ ± 0.1	10.30 ^Bb^ ± 0.3	10.05 ^BCbc^ ± 0.2	9.77 ^CDbc^ ± 0.3	9.46 ^Dbc^ ± 0.3
	30%	11.10 ^Aa^ ± 0.3	10.15 ^Bb^ ± 0.2	9.92 ^BCc^ ± 0.2	9.64 ^Cc^ ± 0.1	9.29 ^Dc^ ± 0.1

± standard deviation. ^A–D^—Same line followed by different uppercase represents a significant difference (*p* ≤ 0.05). ^a–c^—Same column followed by different lowercase represents a significant difference (*p* ≤ 0.05).

**Table 6:** Changes of anthocyanin content (mg/100 g) during storage of blueberry smoothie depending on the addition of yogurt.

Anthocyanins	Addition of Yogurt	Storage Time (Week)				
		0	1	2	3	4
delphinidin derivatives ^1^	0%	5.36 ^Aa^ ± 0.1	5.29 ^ABa^ ± 0.0	5.24 ^BCa^ ± 0.0	5.19 ^Ca^ ± 0.0	5.16 ^Ca^ ± 0.0
	10%	5.38 ^Aa^ ± 0.1	5.29 ^ABa^ ± 0.0	5.22 ^BCa^ ± 0.0	5.17 ^Ca^ ± 0.0	5.16 ^Ca^ ± 0.0
	20%	5.30 ^Aa^ ± 0.1	5.21 ^ABab^ ± 0.1	5.14 ^BCab^ ± 0.0	5.08 ^CDab^ ± 0.0	5.03 ^Db^ ± 0.0
	30%	5.24 ^Aa^ ± 0.1	5.15 ^ABb^ ± 0.0	5.07 ^BCb^ ± 0.0	5.00 ^CDb^ ± 0.0	4.93 ^Db^ ± 0.0
cyanidin and peonidin derivatives ^2^	0%	2.79 ^Aa^ ± 0.03	2.76 ^ABa^ ± 0.04	2.73 ^ABa^ ± 0.03	2.71 ^Ba^ ± 0.02	2.71 ^Ba^ ± 0.01
	10%	2.74 ^Aa^ ± 0.04	2.71 ^Aa^ ± 0.03	2.68 ^ABa^ ± 0.04	2.68 ^ABa^ ± 0.03	2.64 ^Bab^ ± 0.05
	20%	2.66 ^Aa^ ± 0.05	2.61 ^ABb^ ± 0.02	2.56 ^BCb^ ± 0.04	2.52 ^Cb^ ± 0.03	2.50 ^Cc^ ± 0.06
	30%	2.80 ^Aa^ ± 0.09	2.73 ^ABa^ ± 0.05	2.68 ^Ba^ ± 0.02	2.64 ^CBa^ ± 0.03	2.61 ^Cb^ ± 0.01
petunidin derivatives ^3^	0%	4.13 ^Aa^ ± 0.03	4.10 ^ABa^ ± 0.04	4.06 ^Ba^ ± 0.02	4.03 ^Ba^ ± 0.03	4.03 ^Ba^ ± 0.03
	10%	4.09 ^Aa^ ± 0.03	4.05 ^ABab^ ± 0.06	4.00 ^Bab^ ± 0.01	3.98 ^Bab^ ± 0.03	3.96 ^Ba^ ± 0.04
	20%	4.16 ^Aa^ ± 0.04	4.11 ^ABa^ ± 0.03	4.06 ^BCa^ ± 0.02	4.03 ^BCa^ ± 0.01	3.99 ^Ca^ ± 0.03
	30%	4.07 ^Aa^ ± 0.06	4.01 ^ABb^ ± 0.04	3.95 ^ABb^ ± 0.03	3.91 ^BCb^ ± 0.03	3.88 ^Cb^ ± 0.03
malvidin derivatives ^4^	0%	11.88 ^Aa^ ± 0.2	11.39 ^ABa^ ± 0.1	11.31 ^BCa^ ± 0.2	11.26 ^Ca^ ± 0.0	11.24 ^Ca^ ± 0.0
	10%	11.50 ^Aab^ ± 0.0	11.35 ^Ba^ ± 0.0	11.27 ^BCa^ ± 0.0	11.22 ^CDab^ ± 0.0	11.16 ^Db^ ± 0.0
	20%	11.53 ^Aab^ ± 0.1	11.35 ^Ba^ ± 0.0	11.24 ^Ca^ ± 0.0	11.17 ^CDab^ ± 0.0	11.15 ^Db^ ± 0.0
	30%	11.40 ^Ab^ ± 0.0	11.39 ^Aa^ ± 0.1	11.23 ^Ba^ ± 0.0	11.12 ^Cb^ ± 0.0	11.02 ^Dc^ ± 0.0
acylated anthocyanins	0%	4.42 ^Aa^ ± 0.06	4.39 ^Aa^ ± 0.01	4.40 ^Aa^ ± 0.04	4.38 ^Aa^ ± 0.02	4.38 ^Aa^ ± 0.05
	10%	4.37 ^Aa^ ± 0.12	4.24 ^ABb^ ± 0.05	4.22 ^ABb^ ± 0.06	4.19 ^Bb^ ± 0.03	4.19 ^Bb^ ± 0.03
	20%	4.41 ^Aa^ ± 0.03	4.37 ^ABa^ ± 0.03	4.35 ^BCa^ ± 0.02	4.33 ^BCa^ ± 0.03	4.31 ^Ca^ ± 0.02
	30%	4.33 ^Aa^ ± 0.03	4.29 ^ABb^ ± 0.03	4.26 ^BCb^ ± 0.02	4.23 ^Cb^ ± 0.00	4.21 ^Cb^ ± 0.02
Total	0%	28.38 ^Aa^ ± 0.3	27.92 ^ABa^ ± 0.2	27.73 ^BCa^ ± 0.0	27.58 ^CDa^ ± 0.2	27.52 ^Da^ ± 0.2
	10%	28.08 ^Aa^ ± 0.2	27.63 ^Bab^ ± 0.2	27.39 ^BCab^ ± 0.0	27.24 ^CDab^ ± 0.1	27.13 ^Dab^ ± 0.2
	20%	28.06 ^Aa^ ± 0.2	27.65 ^Bab^ ± 0.1	27.36 ^BCab^ ± 0.1	27.13 ^CDab^ ± 0.2	26.98 ^Dab^ ± 0.2
	30%	27.84 ^Aa^ ± 0.2	27.57 ^Bb^ ± 0.0	27.18 ^Cb^ ± 0.2	26.90 ^CDb^ ± 0.1	26.64 ^Db^ ± 0.3

± standard deviation. ^1^ Delphinidin derivatives: delphinidin-3-O-galactoside, delphinidin-3-O-glucoside, delphinidin-3-O-arabinoside. ^2^ Cyanidin and peonidin derivatives: cyanidin-3-O-galactoside, cyanidin-3-O-glucoside, cyanidin-3-O-arabinose, peonidin-3-O-galactoside, peonidin-3-O-glucoside. ^3^ Petunidin derivatives: petunidin-3-O-galactoside, petunidin-3-O-glucoside, petunidin-3-O-arabinoside. ^4^ Malvidin derivatives: malvidin-3-O-galactoside, malvidin-3-O-glucoside, malvidin-3-O-arabinoside. ^A–D^—Same line followed by different uppercase represents a significant difference (*p* ≤ 0.05). ^a–c^—Same column followed by different lowercase represents a significant difference (*p* ≤ 0.05).

## Data Availability

The data used to support the findings of this study can be made available by the corresponding author upon request.

## Supplementary Materials

The following supporting information can be downloaded at: https://www.mdpi.com/article/10.3390/foods12203858/s1, Figure S1: HPLC chromatogram of anthocyanins in strawberry purified extracts in different pH (3.0, 3.5, and 4.5) after 4 weeks of cold storage; Figure S2: HPLC chromatogram of anthocyanins in raspberry purified extracts in different cell-free extracts’ presence after 4 weeks of cold storage; Figure S3: HPLC chromatogram of anthocyanins in blueberry purified extracts with the addition of various doses of hydrogen peroxidase (0; 3.0 and 6.0 µg/mL) after 4 weeks of cold storage. Table S1: Changes in pH during cold storage of smoothies; Table S2: Protein and fat content and the population of lactic acid bacteria in yogurt is used in the production of smoothies.
