# Supplementary material for: Effect of Yogurt Addition on the Stability of Anthocyanin during Cold Storage of Strawberry, Raspberry, and Blueberry Smoothies
Source: Foods. 2023 Oct 21;12(20):3858. doi: 10.3390/foods12203858 (PMC10606227; doi:10.3390/foods12203858)
Supplement: Supplementary file 1 [file foods-12-03858-s001.zip › foods-2666880-supplementary.pdf]

Table S1. Changes in pH during cold storage of smoothies.

Type of smoothie	Addition of yogurt	pH				
		Storage time [week]				
		0	1	2	3	4
Strawberry smoothie	0%	3.46^{Ad}±0.02	3.46^{Ad}±0.00	3.45^{Ac}±0.01	3.46^{Ac}±0.02	3.44^{Ac}±0.01
	10%	3.50^{Ac}±0.02	3.51^{Ac}±0.04	3.51^{Ab}±0.03	3.51^{Ab}±0.02	3.50^{Ab}±0.03
	20%	3.61^{Ab}±0.02	3.58^{ABb}±0.01	3.59^{ABa}±0.01	3.57^{Ba}±0.01	3.57^{Ba}±0.00
	30%	3.67^{Aa}±0.02	3.65^{Aa}±0.03	3.63^{ABa}±0.02	3.60^{Ba}±0.02	3.60^{Ba}±0.01
Raspberry smoothie	0%	3.35^{Ac}±0.03	3.35^{Ac}±0.01	3.34^{Ab}±0.03	3.35^{Ab}±0.03	3.34^{Ab}±0.02
	10%	3.37^{Abc}±0.02	3.37^{Abc}±0.02	3.34^{Bb}±0.01	3.33^{Bb}±0.00	3.34^{Bb}±0.01
	20%	3.41^{Ab}±0.02	3.39^{ABb}±0.03	3.36^{Bb}±0.01	3.35^{Bb}±0.01	3.35^{Bb}±0.00
	30%	3.49^{Aa}±0.01	3.45^{BCa}±0.02	3.42^{Ca}±0.04	3.41^{Ca}±0.03	3.40^{Ca}±0.03
Blueberry smoothie	0%	3.53^{Ad}±0.02	3.54^{Ac}±0.01	3.54^{Ac}±0.01	3.53^{Ac}±0.01	3.54^{Ac}±0.01
	10%	3.61^{Ac}±0.03	3.57^{ABc}±0.03	3.54^{BCc}±0.04	3.48^{CDd}±0.03	3.42^{Dd}±0.03
	20%	3.78^{Ab}±0.04	3.72^{Bb}±0.01	3.70^{BCb}±0.03	3.62^{CDb}±0.04	3.60^{Db}±0.01
	30%	3.92^{Aa}±0.03	3.88^{ABa}±0.02	3.82^{BCa}±0.04	3.78^{CDa}±0.01	3.73^{Da}±0.04

$^{A-E}$ – Same line followed by different uppercase represents a significant difference (p≤0.05).
$^{a-c}$ - Same column followed by different lowercase represents a significant difference (p≤0.05).
± standard deviation

Table S2. Protein and fat content and the population of lactic acid bacteria in yogurt is used in the production of smoothies.

Parameters	Yogurt
Protein content [%]	3.26±0.21
Fat content [%]	3.2±0.17
L. delbrueckii subsp. *bulgaricus* population [log CFU/g]	6.1±0.1
S. thermophilus population [log CFU/g]	8.3±0.1

± standard deviation

Figure S1 HPLC chromatogram of anthocyanins in strawberry purified extracts in different pH (3.0, 3.5, and 4.5) after 4 weeks of cold storage.

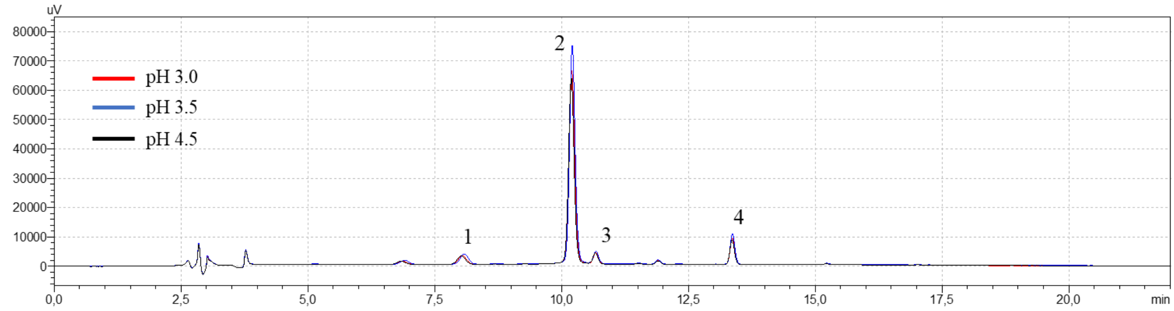

1.Cyanidin-3-O-glucoside, 2. Pelargonidin-3-O-glucoside, 3. Pelargonidin-3-O-rutinoside, 4. Pelargonidin 3-malonyl-glucoside.

Figure S2 HPLC chromatogram of anthocyanins in raspberry purified extracts in different cell-free extracts presence after 4 weeks of cold storage.

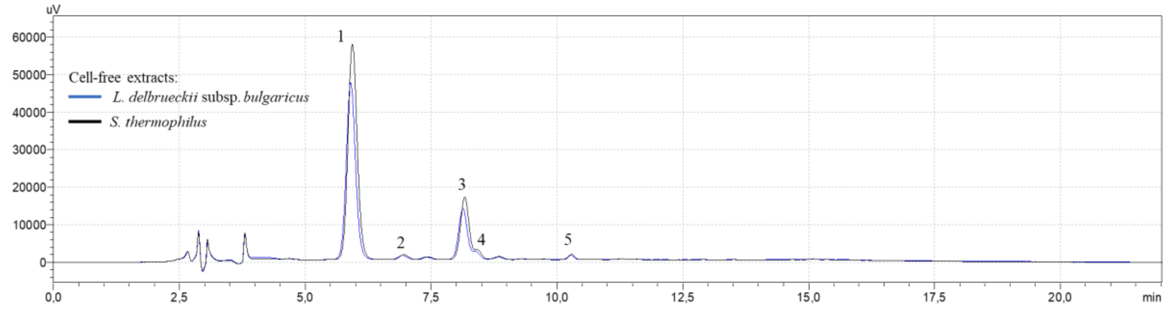

1.Cyanidin-3-O-sophoroside, 2. Cyanidin-3-O-glucosylrutinoside, 3. Cyanidin-3-O-glucoside, 4. Cyanidin-3-O-rutinoside, 5. Pelargonidin-3-O-glucosylrutinoside.

Figure S3 HPLC chromatogram of anthocyanins in blueberry purified extracts with the addition of various doses of hydrogen peroxidase (0; 3.0 and 6.0 µg/mL) after 4 weeks of cold storage.

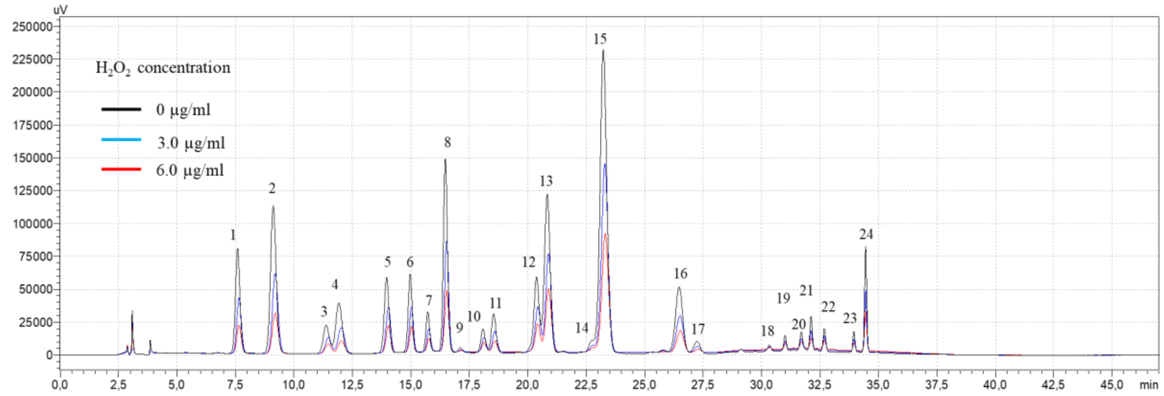

1.Delphinidin-3-O-galactoside, 2. Delphinidin-3-O-glucoside, 3. Cyanidin-3-O-galactoside, 4. Delphinidin-3-O-arabinoside, 5. Cyanidin-3-O-glucoside, 6. Petunidin-3-O-galactoside, 7. Cyanidin-3-O-arabinose, 8. Petunidin-3-O-glucoside, 9. Peonidin-3-O-galactoside, 10. Peonidin-3-O-glucoside, 11. Petunidin-3-O-arabinoside, 13. Malvidin-3-O-galactoside, 15. Malvidin-3-O-glucoside, 16. Malvidin-3-O-arabinoside, 12, 14, 16-24 – acylated anthocyanins.
